# Radiation-Induced Lung Injury With Lung Cancer Treated With the Combination Therapy of Nintedanib and Dexamethasone

**DOI:** 10.7759/cureus.45678

**Published:** 2023-09-21

**Authors:** Junko Itano, Goro Kimura, Kiichiro Ninomiya, Yasushi Tanimoto

**Affiliations:** 1 Department of Allergy and Respiratory Medicine, National Hospital Organization Minami-Okayama Medical Center, Tsukubo-gun, JPN; 2 Department of Allergy and Respiratory Medicine, Okayama Univerity Hospital, Okayama, JPN

**Keywords:** stereotactic body radiation therapy, lung cancer, pulmonary fibrosis, nintedanib, radiation-induced lung injury

## Abstract

Radiation-induced lung injury (RILI) associated with lung cancer becomes refractory. Nintedanib is a multi-kinase inhibitor that suppresses the development of pulmonary fibrosis. Herein, we report a case of RILI with progressive pulmonary fibrosis after stereotactic body radiation therapy in a 70-year-old man with lung cancer. The patient responded well to the initial prednisolone therapy but became resistant during tapering. The combination therapy of nintedanib and dexamethasone resulted in a temporary improvement in RILI. Nintedanib is not a standard therapy for RILI, and further investigation is needed to evaluate the effects of nintedanib on RILI complicated by lung cancer.

## Introduction

Radiation therapy is an essential treatment for lung cancer; however, it can cause severe complications such as radiation-induced lung injury (RILI) with late-onset and progressive pulmonary fibrosis [1,2]. In RILI, the pathogenesis leading to persistent inflammation and fibrosis is promoted by vascular endothelial growth factor (VEGF), fibroblast growth factor (FGF), and platelet-derived growth factor (PDGF) [3] similar to that in idiopathic pulmonary fibrosis (IPF) [4] and chronic progressive fibrosis-interstitial lung disease (F-ILD) [5-7]. The anti-fibrotic role of nintedanib involves inhibiting the binding to the respective receptors for VEGF, FGF, and PDGF, and its anti-inflammatory effects are achieved by regulating pro-inflammatory cytokines and chemokines [8].

Herein, we report a case of RILI with progressive pulmonary fibrosis administrated with a combination of nintedanib and dexamethasone. This investigation was approved by the Research Ethics Committee of the National Hospital Organization, Minami-Okayama Medical Center (# 2022-35). Written informed consent was obtained from the patient and his family to publish the case report.

## Case presentation

**Figure 1:**
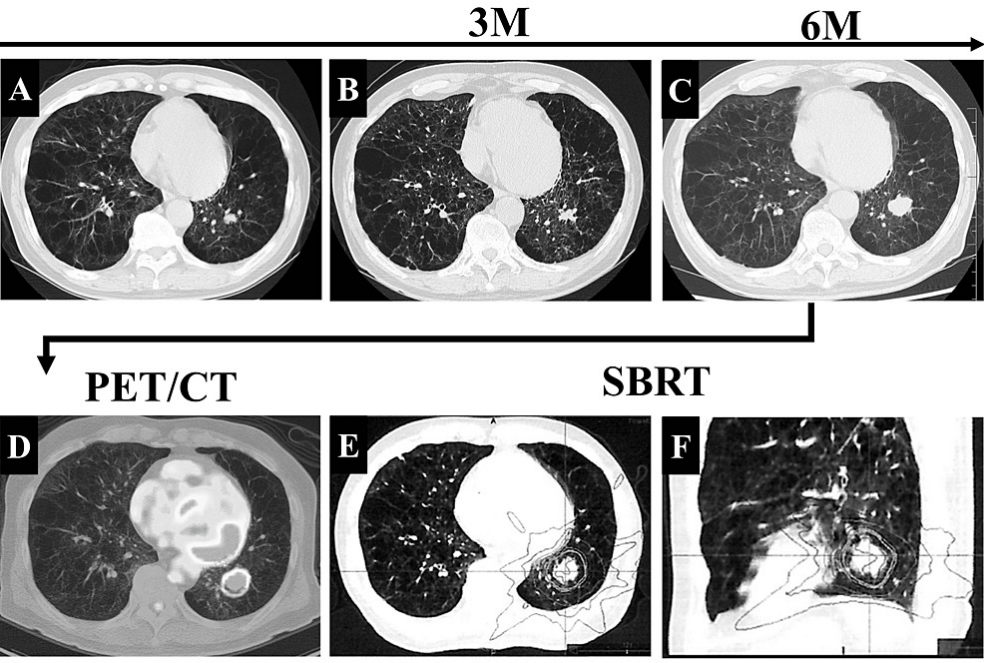
Chest images at the diagnosis and irradiation fields by SBRT. (A) HRCT image of the initially detected nodule. (B, C) Chest CT image three months and six months later. (D) PET/CT scan revealed fluorodeoxyglucose uptake with a maximum standardized value of 20.4. (E, F) Irradiation fields of SBRT. M, months; PET/CT, positron emission tomography/computed tomography; SBRT, stereotactic body radiation therapy; HRCT, high-resolution computed tomography.

A 70-year-old man with a smoking history of 20 pack-years for 34 years and chronic obstructive pulmonary disease presented with a productive cough. He had been on home oxygen therapy for over 10 years and required 4 L/min of oxygen at rest. Medical history included diabetes, hypertension, and pulmonary non-tuberculous mycobacterial infection without relapse 10 years after multidrug chemotherapy. *Pseudomonas aeruginosa* colonized his airways, and he was hospitalized several times with bacterial pneumonia. High-resolution computed tomography (HRCT) revealed an irregular nodule 1.3 cm in diameter in the left lower lung that gradually enlarged, and lung cancer was suspected (Figure 1A, 1B). He underwent a bronchoscopy at another hospital; however, his respiratory condition worsened, and the examination was interrupted. Therefore, the histological diagnosis could not be obtained. We carefully monitored the patient as an outpatient. Six months later, the nodule had grown to 2.5 cm in diameter (Figure 1C), and positron emission tomography/CT (PET/CT) revealed fluorodeoxyglucose uptake (Figure 1D). The HRCT and PET/CT findings suggested the progressive growth of the tumor, and there was no metastasis. We discussed the patient's diagnosis with respiratory physicians, surgeons, and radiologists. As a result, our consensus was that his diagnosis was clinical stage IA3 (T1cN0M0) lung cancer. His Eastern Cooperative Group Performance Status (PS) was 2, and his respiratory function was poor and unmeasurable. We discussed the treatment for lung cancer with the radiologists and performed stereotactic body radiation therapy (SBRT) using CyberKnife (48 Gy in 4 fractions) according to the irradiation fields (Figure 1E, 1F).

**Figure 2:**
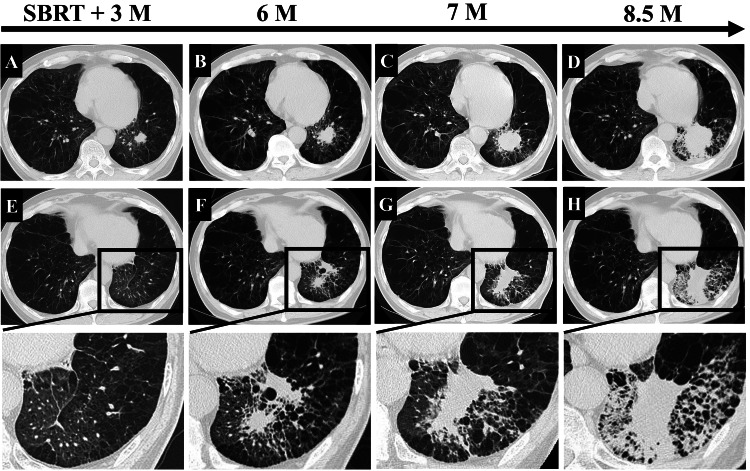
HRCT images after SBRT. (A) HRCT findings showed the reduction of tumors after three months. (B–D) The tumor enlarged gradually again. (E–H) HRCT findings of the development of RILI. Magnified views of the area (bottom) are enclosed by the squares (top). (E) There are no RILI lesions. (F–H) HRCT findings are shown in six months, seven months, and eight and half months after SBRT, respectively. Ground-glass opacities, reticular shadows, and fibrotic changes in the left lower lobe of the irradiated area increased. The development of RILI with fibrotic change was progressive during 8.5 months after SBRT. M, months; HRCT, high-resolution computed tomography; RILI, radiation-induced lung injury; SBRT, stereotactic body radiation therapy.

Although the tumor volume reduced three months after SBRT (Figure 2A), regrowth was observed after six months (Figure 2B-2D). We concluded that the antitumor drugs for lung cancer were not tolerated because the patient's PS was poor. We counseled the patient and his family, and they denied further treatment.

HRCT findings also showed consolidation with ground-glass opacities (GGOs), reticular shadows, and fibrotic change at the irradiation field (Figure 2F). Furthermore, we detected the progressive fibrotic change from 3 to 8.5 months after SBRT (Figure 2E-2H).

Eight-and-a-half months after SBRT, the patient experienced cough and dyspnea. His body temperature was 36.3°C, pulse rate 103/min, blood pressure 144/85 mmHg, and peripheral oxygen saturation (SpO_2_) 94% under 6 L/min oxygen therapy. Fine crackles were heard in his left lungs. Laboratory results upon admission revealed a white cell count of 10,800/mm^3^ (reference range 3500-9100/mm^3^) with 76.6% neutrophils (reference range 35-71.0%), C-reactive protein 4.77 mg/dL (reference range 0.0-0.3 mg/dL), lactate dehydrogenase 152 U/L (reference range 119-229 U/L), carcinoembryonic antigen 1.2 ng/mL (reference range 0.0-5.00 ng/mL), cytokeratin-19 fragment 5.9 ng/mL (reference range 0.0-3.5 ng/mL), squamous cell carcinoma antigen 4.0 ng/mL (reference range 0.0-2.0 ng/mL), Krebs von den Lungen-6 328 U/mL (reference range 0.0-450 U/mL), and surfactant protein D 129 ng/mL (reference range 0.0-109 ng/mL). A nasal swab for severe acute respiratory syndrome coronavirus 2 reverse transcription-polymerase chain reaction was negative. Sputum culture showed only *P. aeruginosa* and no acid-fast bacilli. Sputum cytology was negative.

**Figure 3:**
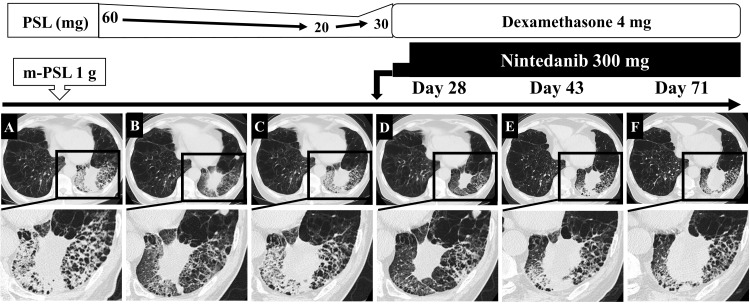
The clinical course and HRCT findings. Magnified views (bottom) of the areas enclosed by squares (top) are shown. (A) HRCT images showing the initial diagnosis of RILI, and GGOs, reticular shadows, and fibrotic changes were detected in the left lower lung of the irradiated area 8.5 months after SBRT. (B) After methyl-prednisolone pulse therapy, the GGOs with the RILI area were reduced. (C) After steroid tapering, at a dose of 20 mg/day, RILI with fibrosis exacerbated and became resistant to steroid therapy. (D) HRCT showed improvement in GGO 28 days after combination therapy of nintedanib (300 mg/day) with dexamethasone (4 mg/day). (E, F) The areas of GGO and consolidation gradually increased again. M, months; HRCT, high-resolution computed tomography; RILI, radiation-induced lung injury; GGOs, ground-glass opacities; SBRT, stereotactic body radiation therapy; m-PSL, methyl-prednisolone; PSL, prednisolone.

HRCT revealed consolidations, GGOs, and reticular shadows, which indicate fibrotic changes in the irradiated area, particularly around the basal segments of the left lower lung (Figure 2H and Figure 3A). Lung injury was diagnosed as RILI, with a grade 3 toxicity grade, according to the Radiation Therapy Oncology Group [1,2]. We started steroid pulse therapy with 1 g of methyl-prednisolone (m-PSL) intravenously for three days. We considered the complication of bacterial pneumonia by *P. aeruginosa* and administrated cefepime (1 g twice daily) for nine days. As shown in Figure 3, after m-PSL pulse therapy, 60 mg/day of prednisolone (PSL) was administered intravenously for two weeks, tapered to 10 mg/day every two weeks (50 mg/day for one week only), and then to 20 mg/day orally.

He responded well to steroids; HRCT findings showed improved consolidations and GGOs (Figure 3B). Two weeks after tapering the steroid to 20 mg/day, he complained of anorexia and dyspnea, and we increased the PSL dose to 30 mg/day. Only normal bacterial flora was detected via sputum culture, and sputum cytology was negative. HRCT revealed the development of GGOs and reticular shadows, which indicated progressive pulmonary fibrosis (Figure 3C).

We considered combination therapy with nintedanib and dexamethasone because the RILI with fibrotic change was progressive and resistant to steroids (PSL), as shown (Figure 2E-2H and Figure 3A-3C). His dyspnea and anorexia persisted, so we carefully counseled the patient and his family, and they requested a combination therapy of nintedanib and dexamethasone. We initiated treatment with dexamethasone at 4 mg/day and nintedanib at 200 mg/day for one week and up to 300 mg/day. His dyspnea and anorexia improved gradually, HRCT imaging showed improvement 28 days after starting combination therapy of nintedanib and dexamethasone (Figure 3D), his dyspnea and anorexia improved, and SpO_2_ could be maintained above 95% with oxygen therapy at 4 L/min at rest. However, HRCT showed the exacerbation of consolidations, reticular shadows, and GGOs (Figure 3E, 3F). After the time point of Figure 3F, the primary tumor in the left lower lung enlarged and was associated with obstructive pneumonia, and his respiratory failure worsened. We carefully counseled him and his family, and they denied any treatment, including antimicrobial agents. The patient died of respiratory failure 83 days after initiating nintedanib.

## Discussion

Radiation induces DNA damage and the release of free radicals, which induce oxidative stress and inflammatory responses [1,2]. Vascular and alveolar cell damage is repeated by pro-inflammatory cytokines and growth factors (e.g., VEGF, FGF, and PDGF), resulting in RILI with irreversible fibrosis [1,9]. Nintedanib targets the receptors of these three growth factors, thereby inhibiting lung inflammation and fibrosis in IPF and other progressive F-ILD [7].

Based on these findings, nintedanib may effectively treat RILI complicated by progressive fibrosis. Nintedanib inhibits fibrosis in the RILI model in vivo [10]. Clinical trials are currently underway to evaluate the efficacy of nintedanib in RILI [11,12]. In a study by Dy GK, nintedanib for patients with non-small-cell lung cancer (NSCLC) after concurrent chemoradiation therapy was terminated owing to accrual logistics; therefore, efficacy could not be evaluated with a small sample size [11]. However, another study showed a significantly lower exacerbation-free rate in patients who added nintedanib to PSL (72%, 95% confidence interval [CI]: 54-96% vs. 42%, 95% CI: 20-82%; P = 0.037) [12]. The pathogenesis of RILI and these data suggest that nintedanib affected the RILI with progressive fibrotic change. In the present case, adding nintedanib to steroids reduced the development of RILI with progressive pulmonary fibrosis.

The limitation of this case study is that we could not perform the pathological assessment. We could not perform a tissue biopsy of the primary tumor and RILI due to respiratory failure and poor PS. However, the HRCT finding showed the development of consolidation with fibrotic change at the irradiated area after three months of SBRT and showed a good response to the first steroid therapy. These HRCT imaging findings and clinical course suggest that the lesion is refractory RILI with active inflammation and fibrosis. The fibrotic changes were progressive, belonging to the progressive F-ILD phenotype [13,14]. Thus, nintedanib therapy was initiated. Moreover, 28 days after starting nintedanib, consolidation, GGOs, and reticular shadows are reduced without antibiotics or anticancer drugs. Although dexamethasone may have ameliorated the RILI, the lesion showed steroid resistance under PSL 30 mg, indicating that dexamethasone alone therapy could not reduce the development of RILI. These data suggest that the additional therapy of nintedanib temporarily improved RILI. However, to our knowledge, no reports indicate clinical and imaging outcomes with nintedanib in RILI with refractory fibrotic change.

In our case, the HRCT findings showed lesions with progressive fibrosis; while treatment with nintedanib may be considered in such cases, nintedanib should not generally be used for RILI. Considering that the administration of nintedanib for RILI itself is not indicated for use by insurance, caution is warranted. Furthermore, nintedanib treatment for RILI is not generally recommended because we could not perform the histologic analysis of lung cancer.

Herein, we presented a case of RILI with active inflammation and progressive fibrosis after SBRT. Combination therapy with nintedanib and dexamethasone ameliorated RILI, which had become steroid-resistant. The effect of nintedanib on RILI is still unknown. However, as mentioned above, future investigations of the therapeutic effect of nintedanib on RILI may be necessary, considering its anti-fibrotic and anti-inflammatory effects.

## Conclusions

The combination therapy with nintedanib and dexamethasone temporarily improved RILI, but histological analysis of lung cancer could not be performed. Nintedanib is not a standard therapy for RILI, and further investigation is needed to evaluate the effect of nintedanib on RILI complicated by lung cancer.
